# Biological exploration of a novel 1,2,4-triazole-indole hybrid molecule as antifungal agent

**DOI:** 10.1080/14756366.2019.1705292

**Published:** 2020-01-03

**Authors:** Fabrice Pagniez, Nicolas Lebouvier, Young Min Na, Isabelle Ourliac-Garnier, Carine Picot, Marc Le Borgne, Patrice Le Pape

**Affiliations:** a Département de Parasitologie et Mycologie Médicale, Université de Nantes, Nantes Atlantique Universités, EA1155 - IICiMed, Institut de Recherche en Santé 2, Nantes, France; b Département de Chimie Thérapeutique, Université de Nantes, Nantes Atlantique Universités, EA1155 - IICiMed, Institut de Recherche en Santé 2, Nantes, France; c Institut des Sciences Exactes et Appliquées (ISEA) - EA 7484, Université de la Nouvelle-Calédonie, Noumea Cedex, New Caledonia; d EA 4446 Bioactive Molecules and Medicinal Chemistry, Faculté de Pharmacie - ISPB, SFR Santé Lyon-Est CNRS UMS3453 - INSERM US7, Université de Lyon, Université Claude Bernard Lyon 1, Lyon, France

**Keywords:** Triazole-indole hybrid, *Candida* species, ergosterol production, phospholipase A2-like activity, *in vivo* evaluation

## Abstract

(2-(2,4-Dichlorophenyl)-3-(1*H*-indol-1-yl)-1-(1,2,4-1*H*-triazol-1-yl)propan-2-ol (**8 g**), a new 1,2,4-triazole-indole hybrid molecule, showed a broad-spectrum activity against *Candida,* particularly against low fluconazole-susceptible species. Its activity was higher than fluconazole and similar to voriconazole on *C. glabrata* (MIC_90_ = 0.25, 64 and 1 µg/mL, respectively), *C. krusei* (MIC_90_ = 0.125, 64 and 0.125 µg/mL, respectively) and *C. albicans* (MIC_90_ = 0.5, 8 and 0.25 µg/mL, respectively). The action mechanisms of **8 g** were also identified as inhibition of ergosterol biosynthesis and phospholipase A2-like activity. At concentration as low as 4 ng/mL, 8g inhibited ergosterol production by 82% and induced production of 14a-methyl sterols, that is comparable to the results obtained with fluconazole at higher concentration. **8 g** demonstrated moderate inhibitory effect on phospholipase A2-like activity being a putative virulence factor. Due to a low MRC5 cytotoxicity, this compound presents a high therapeutic index. These results pointed out that **8 g** is a new lead antifungal candidate with potent ergosterol biosynthesis inhibition.

## Introduction

During the last decades, the frequency of fungal infections has increased because of human immunodeficiency virus infection and more intensive and cytotoxic chemotherapies^1^. In AIDS, oncology and transplantation patients, *Candida* still one of the major opportunistic pathogens^2^^,^^3^. Options for treatment of severe fungal infections are primarily amphotericin B, azole compounds and echinocandins^4^. Although amphotericin B and its lipid formulations decrease the morbidity and mortality, they must be given intravenously, are extremely expensive and still carry significant infusion-related toxicity. Echinocandins, one of the first-line therapy in neutropenic patients reveal a high antifungal activity against *Candida spp*^5^. However, resistance emergence has been described for *C. albicans* and the haploïd yeast *C. glabrata*^6^. Furthermore, elevated MICs have been reported in the case of *C. parapsilosis*.

Fluconazole and fourth generation of azoles (e.g. voriconazole, posaconazole and isavuconazole) remain the most frequently used drugs for treatment of *Candida* infections^7^. However, azole resistance of *C. albicans* and non-*albicans Candida,* such as *C. krusei and C. glabrata,* naturally resistant or low-sensitive to fluconazole, have been reported^8^^,^^9^. Even if new targets have been successfully explored as well as cell wall formation^10^, ergosterol synthesis inhibition has been the major objective of new antifungal drugs being ravuconazole and isavucoanzole the most recent clinically available drugs. Most of new antifungal targets presented in the literature exhibited little potential to develop target-based inhibitors being pharmacomodulation of non-toxic azole a good alternative^11^.

Indolylazole derivatives are a new class of azole antifungal drugs^12^ which have demonstrated *in vitro* activities against *Aspergillus*^13^ and protozoa such as *Leishmania*^14^^,^^15^. More recently, structural modifications were done to enable a specific and broad activity against *Candida* spp., including fluconazole low-susceptible species as *C. krusei* and *C. glabrata*.

*Candida* spp. are known to produce several types of phospholipases such as phospholipase B and phospholipase A which were detected in culture supernatant^16^^,^^17^. The virulence of *Candida* strains in *G. mellonella* is related to the quantity of phospholipases production^18^ and strains isolated from symptomatic women with vulvovaginal candidiasis exhibit high level of phospholipase activity^19^. Phospholipase B has been described as a pathogenic factor that contributes to host membrane degradation and penetration of *C. albicans* pseudohyphae^20^. Intracellular phospholipases, identified as phospholipases A, have been localised at the site of bud formation^21^. Triglyceride lipases with phospholipase A2-like activity (patatin-like homologs) have been described in *Saccharomyces* and *Candida*^22^. Already known function of this phospholipase is hydrolysis of fatty acids for membrane formation and sporulation. Since indole derivatives have been reported as inhibitors of phospholipase A_2_ activity^10^^,^^23^ our 2-dichlorophenyl-3-triazolyl-1-indolyl-propan-2-ol derivative (**8 g**) could be of interest to inhibit this enzyme activity.

In this work, we present the potent anti-*Candida* activity of **8 g**, an indole-triazole derivative, against *C. albicans* and non-*albicans* clinical isolates and its mechanism of action as inhibitor of ergosterol biosynthesis and phospholipase A2-like activity.

## Materials and methods

### Strains

Control strains used for *in vitro* evaluation were the CLSI reference strains, including *Candida albicans* ATCC 2091, *C. glabrata* CBS 138, *C. krusei* ATCC 6258, *C. parapsilosis* ATCC 22019 and *C. parapsilosis* ATCC 90018. Additionally, numerous clinical isolates from the collection of EA1155 IICiMed were used: *C. albicans* (*n* = 26), *C. glabrata* (*n* = 12), *C. krusei* (*n* = 14), *C. parapsilosis* (*n* = 18) and *C. tropicalis* (*n* = 6). The selected species are representative of the European epidemiology and therefore correspond to the five most prevalent species in the clinic settings^24^. Strains were maintained and subcultured 24 h before used on Sabouraud’s agar slants.

### Chemicals

**Figure 1.**
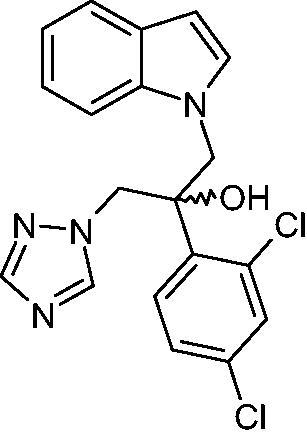
Chemical structure of **8 g**.

Indole-triazole derivative compound **8 g**, (2–(2,4-dichlorophenyl)-3-(1*H*-indol-1-yl)-1–(1,2,4-1*H*-triazol-1-yl)-propan-2-ol (Figure 1), was synthesised in the Department of Medicinal Chemistry, EA1155 IICiMed. The chemical synthesis route of **8 g** and its derivatives (not described in this article) was achieved with regard to the synthesis of new analogues of fluconazole.^25^ **8 g** was dissolved in DMSO to prepare a stock solution at 10 mg/mL. This stock solution was diluted in RPMI 1640 for *in vitro* experiment. For *in vivo* studies, to avoid injecting DMSO, compound **8 g** was prepared in 0.5% Tween 20-sterile saline solution. Fluconazole and voriconazole (Pfizer) were obtained from Mycobiotics. Stock solutions were prepared in water and DMSO, respectively, at a concentration of 10 mg/mL.

### Antifungal susceptibility testing

MICs for *Candida* strains were determined by the broth microdilution method with RPMI 1640 medium (Sigma Aldrich, Saint Quentin fallavier, France) according to the procedures of the Clinical and Laboratory Standard Institute described in document M27-A2^26^. Yeasts were grown on Sabouraud dextrose agar (Difco Laboratories) at 35 °C for 24 h. The wells were inoculated with 100 µL of the culture suspension diluted to a final inoculum of 5 x 10^2^ to 2.5 x 10^3^ cells/mL. Fungal growth was observed 48 h after incubation at 35 °C. The MICs of the azoles were the lowest drug concentrations to give a 50% decrease in turbidity from that of the control fungal growth. MIC_90_ was the concentration of a drug required at which 90% of the clinical isolates tested were inhibited.

### MRC5 toxicity assay

Cytotoxicity of compounds was studied with human fibroblast (MRC5). Cells were grown in RPMI 1640 medium (Sigma-aldrich) supplemented with 10% foetal bovin serum (Sigma-aldrich). Drugs were tested at three concentrations (100, 10 and 1 µM) in triplicate. After a 96 h-incubation time cytotoxicity was measured on the Fluorolite 1000 (Dynatech, France) after a 4-h incubation time with Uptiblue^®^ (Interchim, Montluçon, France). Inhibitory concentration 50 is a mean of triplicate values. A toxicity index was determined as follow: IC_50_ against MRC5/Geometric mean of the MICs against *Candida spp*.

### Phospholipase A_2_ activity

Phospholipase A_2_-like activity (PLA_2_) was quantified in cells and culture supernatant of *C. albicans* CA98001 clinical isolate incubated for 1 h at 37 °C with the specific modified phospholipase A_2_ substrate, 1-octadecanoyl-2–(1-^14^C)-eicosatetraenoyl-glycerol-3-phosphocholine. Lipids were extracted with chloroform/methanol mixture. Aliquots of chloroformic phases were placed in scintillation flasks for measures of total lipid radioactivity. The chromatography solvent phase was toluene/dioxane/acetic acid/formic acid 85/15/0.2/0.2 (v/v). Lipid classes were visualised with iodine vapours on an analytical thin-layer chromatography. Each spot was cut and the lipids were solubilised in scintillation liquid. Then, radioactivity was measured in a LKB 1909 scintillation spectrophotometer^8^. PLA_2_ activity was expressed as the ratio of radioactive fatty acids versus total radioactivity of sample.

Compound **8 g** was introduced 30 min before the radioactive substrate at a concentration of 10 µM. A known inhibitor of PLA2, the 4-bromophenacyl bromide (BpB) was used as reference at a concentration of 10 µM.

### Sterol extraction

To study sterol synthesis, *Candida albicans* CAAL93, *C. krusei* CAKR7 and *C. glabrata* CAGL2 blastopores were incubated in 50 ml Sabouraud broth medium (Sigma) during 18 h at 35 °C with stirring. Cells were collected by centrifugation at 1500 g. Pellet was suspended in 3 ml of fresh ethanolic potassium hydroxide solution (25 g of KOH, 36 ml of distilled water and brought to 100 ml with 100% ethanol). Saponification was performed at 80 °C for 60 min. Sterols were extracted by addition of 3 ml of hexane and the organic phase was then washed by adding 1 ml of sterile water. Final organic phase was transferred to a new collection tube after 5 min centrifugation step at 2000 rpm.

### Sterol analysis

Sterols as TMS derivatives were analysed by GC-MS using an Agilent 6890 N GC system, with a HP-5MS column (30 m x 0.25 mm ID, 0.25 µm film thickness, Agilent Technologies) coupled with a quadrupole mass detector (Agilent 5973i – E.I. 70 eV). Two microliters of sample were injected in splitless mode at 250 °C. The carrier gas was helium at a flow rate of 1.2 mL/min. The oven was set at 150 °C for 0.5 min and then raised to 280 °C at 40 °C/min and from 280 °C to 300 °C at 2 °C/min. Sterols were identified via their electron ionisation fragmentation pattern, compared to published data. Results are expressed as percent area of total sterols.

### *In vivo* activity

#### Animal use and care

Swiss female mice (Janvier Labs, Le Genest-Saint-Isle, France) with a body weight of ∼25 g were obtained and allowed to acclimate for a minimum of 5 days prior to use. Environmental controls for the animal room were set to maintain a temperature of 16 to 22 °C, a relative humidity of 30 to 70%, and a 12:12 hourly light-dark cycle.

#### Systemic C. albicans infection model

Mice (8 per groups) were immunodepressed by subcutaneous injection of 30 mg/kg prednisolone one day before challenge. On day 0, mice were infected intravenously with *C. albicans* (CAAL93) blastoconidia (5.10^5^ in 100 µL of NaCl 0.9%). One hour after infection, mice were treated intraperitoneally for five consecutive days with 100 µL 0.5% Tween 20-sterile saline solution (control), twice daily with 30 mg/kg or three times a day with 20 mg/kg body weight of compound **8 g**. Fluconazole (Sigma, St Quentin Fallavier, France) was administered *per os* three times a day at 5 mg/kg. Mice were monitored for 14 days post inoculation. For reduction of animal distress, the body weights of the mice were recorded daily, and when they lost 20% of their initial weight, mice were euthanized. On day 14, all surviving mice were euthanized.

### Statistical analysis

The results were statistically analysed using the software Graphpad PRISM 7.0. The log-rank (Mantel-Cox) test was performed for survival analysis. A *p* values < 0.05 was considered significant.

## Results

Antifungal activities (MICs) of **8 g** and reference compounds (fluconazole and voriconazole) against our set of clinical *Candida* strains are summarised in Table 1. Against CLSI quality control strains, MICs of **8 g** were 0.125, 0.016 and 0.032 µg/mL for *C. krusei* ATCC6258, *C. parapsilosis* ATCC22019 and *C. parapsilosis* ATCC90018, respectively. This compound displayed high significant *in vitro* activity against the most clinically important species tested especially against low-susceptible or resistant *C. glabrata* and *C. krusei*. Indeed, **8 g** was always more potent than fluconazole and at least as potent as voriconazole. MIC_90_s against *C. glabrata* and *C. krusei* were as low as 0.25 and 0.125 µg/mL, respectively, while MIC_90_s of fluconazole were 64 and > 64 µg/mL, respectively. MIC_90_s of voriconazole were 4 and 4 µg/mL, respectively. MIC_90_s against completely tested strains were 0.5, 64 and 1 µg/mL for **8 g**, fluconazole and voriconazole, respectively.

**Table 1.** Antifungal susceptibilities of *Candida* spp. to **8 g**.

Species (no. of isolates)	Antifungal agent	MIC (µg/mL)		
		Range	Geometric mean	MIC_90_
*C. albicans* (27)	**8g**	< 0.016–4	0.038	0.5
	fluconazole	< 0.125 – > 64	0.696	8
	voriconazole	< 0.016 – > 8	0.042	0.25
*C. glabrata* (13)	**8g**	< 0.06–0.5	0.06	0.25
	fluconazole	0.016 – > 64	4	64
	voriconazole	0.016–4	0.25	1
*C. krusei* (15)	**8g**	0.016–1	0.109	0.125
	fluconazole	8 – > 64	23	64
	voriconazole	0.016–4	0.158	0.125
*C. parapsilosis* (20)	**8g**	< 0.016–0.125	0.029	0.0625
	fluconazole	< 0.125 – > 64	0.661	8
	voriconazole	< 0.016–0.25	0.017	0.125
*C. tropicalis* (6)	**8g**	< 0.016–1		
	fluconazole	< 0.125–64		
	voriconazole	< 0.016–0.5		
*Candida* spp. (81)	**8g**	< 0.016–4	0.048	0.5
	fluconazole	< 0.125 – > 64	2	64
	voriconazole	< 0.016 – > 8	0.059	1

Cytotoxicity evaluated on human fibroblast (MRC5) showed an IC_50_ of 35 µM for **8 g** that is lower than for fluconazole or voriconazole (IC_50 _>100 µM). Nevertheless, its cytoxicity occurred at concentration 280-time higher than geometric mean MIC against *Candida* (while fluconazole and voriconazole toxicity index were > 15 and > 590, respectively).

**Figure 2.**
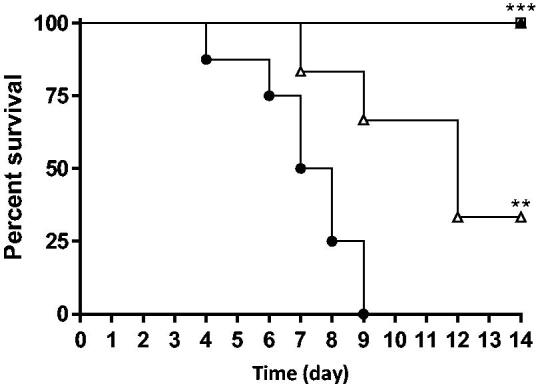
Survival curve of mice after treatment with fluconazole (3 x 5 mg/kg *per os*, □) or **8 g** (2 x 30 mg/kg ip, Δ; 3 x 20 mg/kg ip, ▲). Control group (●). **p < 0.01; ***p <0 .001.

Compound **8 g** was evaluated *in vivo* in a mouse model of systemic candidiasis. Survival of mice was significantly (*p* < 0.01) higher in fluconazole and **8 g** groups than in the control group (Figure 2). In the fluconazole group (3 x 5 mg/kg) and in the **8 g** group (3 x 20 mg/kg), all mice survived. Moreover, a treatment three times a day (3 x 20 mg/kg) was significantly (*p* < 0.05) more effective than twice a day (2 x 30 mg/kg).

A PLA2-like activity was clearly characterised in *C. albicans* CA98001, including cytosolic PLA2 (cPLA2) and secreted PLA2 activities. Indeed, using intact *Candida* cells, 53% and 34% of PLA2 activity was inhibited by 10 µM BpB and **8 g**, respectively (Table 2). In the case of **8 g**, the impact on the production of arachidonic acid is marked (0.12 for **8 g** *versus* 0.22 for the control).

**Table 2.** Hydrolysis inhibition of the 1-octadecanoyl 2–(1-^14 ^C) eicosatetraenoyl glycerol-3-phosphocholine after treatment with bromophenacyl bromide (BpB) and **8 g**.

	Inhibitors (Mean ± SEM)		
	Control	BpB	**8g**
phospholipids	1.18 ± 0.18	1.32 ± 0.01	1.10 ± 0.01
mono- & diglycerides	0.12 ± 0.04	0.12 ± 0.01	0.14 ± 0.02
arachidonic acid	0.22 ± 0.04	0.11 ± 0.01	0.12 ± 0.02
triglycerides & others	0.26 ± 0.13	0.31 ± 0.16	0.12 ± 0.01
total lipids	1.79 ± 0.05	1.86 ± 0.15	1.48 ± 0.04
PLA2 like activity (%)	12.5 ± 2.6	5.9 ± 0.79	8.2 ± 1.06

Radioactivity of the lipid classes is expressed in nmol.L^−1^.min^−1^/10^8^ cells. Phospholipase activity is expressed as the percentage of fatty acid released from the total radiolabelled phospholipids.

GC-MS analysis of sterol profile of three different clinical strains of *C. albicans* (CAAL93), C. *krusei* (CAKR7) and C. *glabrata* (CAGL2) showed ergosterol as the major sterol accounting for 69% to 83% of total cellular sterols. Small quantities of ergosterol intermediates such as zymosterol, ergosta-7,22-dienol, fecosterol and episterol were also detected to a lesser extent (between 2% and 5%). Ergosterol precursor in *Candida*, i.e. lanosterol, was also found in a small quantity (0.7% to 5.2%). These sterol profiles were in accordance with classical metabolism of ergosterol^27^. Treatment with **8 g** (4 ng/mL) of C. *albicans* fluconazole-susceptible strain (CAAL93 – MIC = 0.125 µg/mL) resulted in a significant reduction of ergosterol content (70%), an accumulation of lanosterol and the emerging of 14α-methyl sterols such as eburicol, 14α-methylfecosterol and 14α-methylepisterol. Same modifications in sterol profile were observed when CAAL93 was treated with fluconazole at a 1000-fold higher dose (4 µg/mL) (Table 3).

**Table 3.** Sterol profile of *C. albicans* (CAAL93), *C. krusei* (CAKR7) and *C. glabrata* (CAGL2) untreated and treated with fluconazole (FLU: 4 µg/mL) or **8 g** (4 ng/mL or 4 µg/mL). –: not detected.

		*CAAL93*			*CAKR7*			*CAGL2*		
		control	**8g**	FLU	control	**8g**	FLU	control	**8g**	FLU
	R.T.		4 ng/mL	4 µg/mL		4 µg/mL	4 µg/mL		4 µg/mL	4 µg/mL
*zymosterol*	9.34	4.8	–	–	2.4	–		–	–	–
*24-ethyl-cholesta-5,7,22-trienol*	9.49	–	–	8.5	–	–	–	–	–	–
***ergosterol***	**9.55**	**69.4**	**21.6**	**–**	**83.1**	**60.1**	**84.3**	**78.4**	**41.6**	**79.9**
*14α-methylepisterol*	9.71	–	13.5	30.7	–	3.5		–	2.6	–
*ergosta-7,22-dienol*	9.73	5.5	–	–	–	–	–	–	–	–
*fecosterol*	9.80	–	–	–	1.9	–	1.8	–	–	–
*14α-methylfecosterol*	9.97	–	10.0	10.8	–	4.9		–	3.5	–
*ergostadien-3β-ol (5,7 or 5,8)*	10.05	–	–	–	1.1	–	1.1	3.7	2.2	7.6
*episterol*	10.26	3.4	–	–	–	–	–	1.9	–	3.5
***14****α****-3,6-diol***	**10.41**	**–**	**–**	**–**	**–**			**–**	**23.4**	**–**
***lanosterol***	**10.55**	**5.2**	**35.4**	**34.2**	**0.7**	**24.2**	**1.8**	**3.6**	**–**	**1.9**
*obtusifoliol*	10.60	–	–	–	–	–	–	–	23.8	–
*4,4-dimethylcholesta-8,24-dienol*	10.80	1.4			1.2		1.1	2.7		2.1
*eburicol*	11.20	–	19.0	15.7	–	3.9	–	–	0.7	–
*other sterols*		10.3	0.5	0	9.5	3.5	10.0	9.6	2.2	7.1

R.T.: retention time; Other sterols: sterols <1% or unidentified sterols. Bold: sterols of major interest (lanosterol = precursor, ergosterol = final product and 14a-3,6-diol = toxic final product)

Two fluconazole-resistant strains (CAKR7, MIC ≥ 64 µg/mL and CAGL2 MIC ≥ 64 µg/mL) were treated with 4 µg/mL fluconazole and no effect on sterol profile was observed. However, when CAKR7 and CAGL2 strains were treated with **8 g** at the same dose, a significant diminution of ergosterol content was observed (25% to 47%) as well as an accumulation of lanosterol or obtusifoliol. As for CAAL93, a production of 14α-methyl-sterols was observed: eburicol, 14α-methylfecosterol and 14α-methylepisterol. Toxic 14α-methyl-diol was even detected for CAGL2 (23%). Modifications in sterol profiles after treatment with **8 g** confirmed the blockage of 14α-demethylase (ERG11) by this compound.

## Discussion

Development of new antifungal drugs implies a high level of activity against targeted fungi and acceptable toxicity against host cells. In this aim, we have developed a new family of azole derivatives, indolyl-triazoles^25^, that could inhibit yeast growth at low concentrations (MIC_90_s < 0.50 µg/mL). The compound **8 g** has a broad-spectrum activity against most clinical relevant *Candida* species. Against all *Candida* spp., MIC range of **8 g** (< 0.016 – 4 µg/mL) was narrower than for fluconazole or voriconazole with MIC ranges of < 0.125 – > 64 µg/mL and < 0.016 – > 8 µg/mL, respectively. Against fluconazole low-susceptible spp. (*C. glabrata* and *C. krusei*), **8 g** was more active than fluconazole and voriconazole. Because of their increasing frequency in bloodstream infection and their ability to resist to antifungal drugs, these species are now the real target of new drugs^6^^,^^28^. **8 g** inhibited their growth at very low concentrations (MIC_90_s = 0.25 µg/mL) whereas fluconazole and voriconazole have higher MIC_90_s, 64 and 1 µg/mL, respectively. Moreover *C. albicans*, being the most frequently isolated species during invasive candidiasis, is highly sensitive to **8 g** (MIC_90_ < 0.5 µg/mL). Second-generation triazole antifungals (voriconazole, posaconazole, isavuconazole) have a broad anti-infectious spectrum. When tested against *Aspergillus fumigatus*, **8 g** showed a moderate activity, since the concentration that inhibited 80% of the growth was 23 µg/mL. Because triazole derivatives (itraconazole, fluconazole and posaconazole) could inhibit ergosterol-like synthesis in Trypanosomatids^29^, **8 g** was tested against *Leishmania*. Results (data not shown) showed that **8 g** was effective *in vitro* (IC_50_ = 8 ± 2 µg/mL) against *Leishmania mexicana* promastigote stage. Further investigations must be done to evaluate activity against the most relevant amastigote stage encountered in human host.

*In vivo*, compound **8 g** confirmed its antifungal activity against *C. albicans*. In a systemic candidiasis model, it was as effective as fluconazole to allow mice survival. In addition, the modification of the initial administration protocol (the same dose three-times a day instead of twice a day) gave a complete protection. This second protocol also highlighted the importance to spread out the daily dose of **8 g**, thus providing 24 h of protection against a systemic candidiasis.

Several events are involved in the membrane biosynthesis, especially the production of lipids as sterols and phospholipids. We demonstrated that **8 g** inhibits *Candida* ergosterol synthesis as observed with other azole drugs. This inhibition led to membrane integrity perturbations through ergosterol depletion, precursor accumulation (lanosterol) and production of alternative 14α-methyl sterols (14α-methyl-fecosterol, 14α-methyl-episterol and 14α-methyl-3,6-diol). Identification of 14α-methylated sterols evidenced a blockage of CYP51 as a way of action of **8 g**. Interestingly, the effects of **8 g** on sterol metabolism are observed in CAAL93 (fluconazole susceptible strain) at 4 ng/mL whereas the same results are observed after fluconazole treatment at a 1000-fold higher concentration (4 µg/mL). Moreover, the same effects are seen when fluconazole-resistant strains (CAKR7 and CAGL2) were treated by **8 g** at 4 µg/mL whereas nothing happened after treatment with the same dose of fluconazole. These results show the potency of this new indole-triazole derivative compound.

Phospholipids are vital structural and functional entities of biomembranes. Phosphatidylcholine, the major phospholipid, is in part metabolised by phospholipases A (PLAs). These enzymes are known to be essentials for the turnover of the membrane phospholipids. Pugh *et* *al* showed that PLAs were present at the site of bud formation suggesting that they are involved in growth and change in shape of the cell wall^21^. With a different approach, previously used by Le Pape *et* *al*, we described a sn-2 hydrolytic phospholipase (PLA_2_) activity in *Candida albicans*^30^. After 1-h contact time, this activity was inhibited by **8 g** suggesting an interaction with somatic PLA2-like lipase. Whereas BpB decreased 12% of the glycerophospholipid hydrolysis, **8 g** did not inhibit the secreted phospholipase activity (data not shown). However, no gene coding for PLA2 was identified in yeast but lipases with patatin-like domains which have a cPLA2-like activity, have already been described in *Saccharomyces cerevisiae* and in *C. albicans*^22^^,^^31^. Patatin-like phospholipases role is well established for membrane biogenis and sporulation, but their implication in virulence or pathogenesis had still to be determined. Such enzymes could be future targets for new antifungals. Thus, high **8 g** concentrations (10 µM) could inhibit cell phospholipid organisation and could complete the main mechanism of action, e.g. inhibition of sterol biosynthesis.

Because of moderate toxicity on MRC5 cells but very low MIC against *Candida,* our compound exhibited a good safety (toxicity index = 280) as observed for reference drugs (fluconazole and voriconazole).

In conclusion, **8 g** showed high *in vitro* activity against *Candida*, including low fluconazole-susceptible species (*C. krusei* and *C. glabrata*) which remain one of the main challenges in candidiasis therapy. Its mechanism of action involved a strong blockage of CYP51 and in a minor importance inhibition of PLA_2_-like activity. This promising compound has low toxicity on MRC5 cells and a significant *in* *vivo* activity. Further investigations must be done to evaluate antifungal activity of **8 g** and its enantiomers in *in vivo* models of candidiasis and against other fungi.
